# The Effect of Training on Stride Duration in a Cohort of Two-Year-Old and Three-Year-Old Thoroughbred Racehorses

**DOI:** 10.3390/ani9070466

**Published:** 2019-07-22

**Authors:** Rebecca S. V. Parkes, Renate Weller, Thilo Pfau, Thomas H. Witte

**Affiliations:** 1 Jockey Club College of Veterinary Medicine and Life Sciences, City University of Hong Kong, Kowloon, Hong Kong; 2 Royal Veterinary College, Hatfield, Herts AL9 7TA, UK

**Keywords:** horse, biomechanics, adaptation, gait analysis, training

## Abstract

**Simple Summary:**

Objective gait monitoring via GPS and motion sensors is becoming increasingly popular with racehorse trainers. This has the potential to assist in early detection of lameness and performance issues. This study sought to identify normal changes in gait in a population of two and three-year-old racehorses in order to inform future studies. We found that horses decrease their stride duration at a given speed over time with training. Stride duration appears to increase with increased distance galloped, but this effect is reduced over a training season and presumably increased fitness, so this may serve as a useful indicator for fatigue.

**Abstract:**

Objective gait monitoring is increasingly accessible to trainers. A more comprehensive understanding of ‘normal’ gait adaptations is required. Forty two-year-old thoroughbred racehorses were recruited when entering training and followed for 22 months. Gait analysis was performed by equipping each horse with an inertial measurement unit with inbuilt GPS (GPS-IMU) mounted on the dorsum. Horses were exercised as per their regular training regimen. Data were analysed using a linear mixed model. For two-year-old horses, there was a non-linear pattern of stride duration (SD) over time (*p* < 0.001) with SD decreasing initially and then ‘flattening off’ over time (linear and quadratic coefficients −0.29 ms/week and 0.006 ms/week^2^). Horses showed an increase in SD of 2.21 ms (*p* < 0.001) per 100 m galloped, and over time, SD decreased by 0.04 ms (*p* < 0.001) with each 100 m galloped per week. Three-year-old horses overall showed no change in SD over time (*p* = 0.52), but those that had a period of time off showed a decrease in SD of −0.59 ms per week (*p* = 0.02). They showed an increase in SD of 1.99 ms (*p* < 0.001) per 100 m galloped, and horses that had a period of time off showed an increase in stride duration of 1.05 ms per 100 m galloped (*p* = 0.01) compared to horses which did not have time off. Horses demonstrate an adaptation to high-speed exercise over time. SD decreases with training when other factors are controlled for in naïve horses.

## 1. Introduction

Musculoskeletal injury is a major cause of lost training days in the Thoroughbred [1,2], and most injuries occur during training [3]. Early training appears beneficial, as horses that begin training and race as two-year-olds have more prolonged careers than those entering training at a later date [4,5]. A more comprehensive understanding of the alterations in normal gait during training may help to identify normal gait changes and, thus, recognition of prodromal changes prior to injury [6,7].

Stride parameters may change in response to different circumstances, for example, horses galloping on an incline gallop relatively slower [8] and with a higher stride frequency for a given speed compared with a flat surface [9], with a shift in vertical impulse from the fore to the hindlimbs [9,10]. Protraction duration [11] and the suspension phase of the gallop [12] do not appear to change significantly with speed at a group level, although individual horses display some decrease of their suspension phase with increasing speed [12]. When racing on a curve at the gallop, horses slow down, which may be due to a force limit on the limbs [13].

Some features of gait have been shown to change with training, but studies investigating the effects of training on the gait of Thoroughbreds are limited in their scope, partly due to difficulties in assessing gait at high speed especially in field conditions.

When trotting over ground, a group of seven two-year-old Thoroughbred fillies showed an increase in stride length and stride duration and a decrease in stance duration following training in comparison to untrained individuals [7] and a study of eight mature Thoroughbred geldings training over a five month period increased stride frequency and decreased protraction time at the gallop, but there was no effect on stance duration or stride length [6]. During treadmill locomotion, a group of six Thoroughbred fillies were shown to significantly reduce stance duration and increase overall stride duration at all speeds and to increase the vertical lift of limb segments, but they showed no change in stride length at any given speed [14].

No study has yet assessed the effect of training on stride parameters in a cohort of immature Thoroughbreds training under ’normal’ conditions. We aimed to evaluate gait changes in a cohort of horses over their first training season.

Based on previous studies, we hypothesised that at the gallop:

1. Horses would show a decrease in stride duration over time [7]
2. Responses to different conditions such as galloping on a curve or on an incline would change over time.

## 2. Materials and Methods

### 2.1. Horses

Forty Thoroughbred racehorses entering training at one training facility were convenience sampled for a prospective cohort study. There were twenty-eight fillies, ten colts, and two geldings, aged between 19.4 and 23.0 months (average 21.4 ± 1.1 months) at the start of the study. Data collection began when horses had started the training regimen and had begun canter work. Some horses began basic ‘pre-training’ and were broken to saddle at another centre prior to arriving, but none had begun regular cantering exercise prior to entering the training yard. 

### 2.2. Data Collection

Each horse was equipped with one Global Positioning System-Inertial Measurement Units (in-house design, Structure and Motion Laboratory, Royal Veterinary College, UK). These units contained a GPS unit sampling at 10 Hz, a tri-axial ±20 g accelerometer sampling at 300 Hz and a ±2000 degrees/s gyroscope sampling at 300 Hz. The units were mounted on the dorsal midline immediately caudal to the most caudal palpable dorsal spinous process using a woven elastic self-adhesive porous foam plaster (Polster-Plast, Animal Polster, Vest-Agder, Norway). The units were applied by the same trained individual on each occasion. The sacrum location was chosen on the basis of preliminary trials, as it minimises interference from the jockey and tack, and allows a clear sky-view for the GPS units. Horses underwent routine training with a rider, consisting of a warm-up at walk and trot followed by a variable period of galloping exercise at a speed as defined by the trainer. Exercise took place on a synthetic training surface, in a straight line or on a curve and on a flat or inclined track, as defined by the trainer. The gallops were well maintained and harrowed on a daily basis. Details of expected training speed and jockey were recorded manually, and the location, curve, incline, actual speed, duration of training and stride parameters were recorded by the GPS-IMU. The GPS within the IMU was a commercial unit previously validated for accuracy of speed measurement, although curves of small radius show a slight underestimation of speed [15].

Data collection was performed at intervals on thirteen occasions over eleven months (December 2012 and October 2013). The mean interval between data collection sessions was 3.6 ± 1.6 weeks, and the initial data collection period spanned 43.6 weeks. After October 2013, eighteen horses had a rest period and did not race, instead remaining in light training (walking and trotting) over the winter. Six horses continued to race over the winter season, and as such remained in training. Details of which horses raced over the winter and which did not were recorded to allow comparisons between trained and rested groups.

Data collection resumed in February 2014 as the horses entered their three-year-old year, and was performed on seven occasions over nine months, until the study finished in October 2014. At the start of data collection in February 2014, 24 horses remained in the study. The average interval between data collection sessions was 5.7 ± 2.3 weeks.

### 2.3. Data Analysis

Sample size estimates were performed using G*Power (Heinrich-Heine-Universität, Düsseldorf, Germany). This was based on a comparison of the means of pilot stride duration data from immature and mature Thoroughbreds. Sample size estimates suggested that between 16 and 78 horses would give 80% power with 95% confidence to detect effect sizes of between 0.67 and 0.29 ms change over time. 

Raw GPS data were processed using commercial software (Grafnav Batch v8.2, Waypoint, Calgary, Canada), providing positional data for each horse. IMU and GPS data for each horse were automatically time-matched within the sensors, and GPS data was upsampled from 10 Hz to 300 Hz to avoid any loss of IMU data using custom software (MATLAB R2012b, The Mathworks, Natick, USA). Trial times were automatically selected by identifying periods when the horses were travelling at > 7 m s^−1^.

Trials were segmented into individual strides by identifying the time of peak displacement in a dorso-ventral direction, which has been shown to be accurate for quantifying stride duration in preliminary studies [16]. Horizontal speed for each stride was calculated from raw GPS data, and stride frequency and stride length were calculated using stride duration.

The effective radius of all track segments was calculated using the formulae [13]:

(1) $$\omega =\;\frac{\Delta \theta }{\Delta t}$$

(2) $$ri=\;\frac{v}{\omega }$$

where *ω* is angular velocity, Δ*θ* is change in heading, Δ*t* is stride duration, *r_i_* is effective radius and *v* is velocity. 

Segments with an effective radius of >200m were defined as ‘straight’ and of <200m as ‘curved’. The incline of the track was defined by calculating the mean change in height above sea level over a period of five strides using a moving window in order to account for within-stride fluctuation in the height of the horse. Incline was then categorised as ‘flat’ (−2 to 2% gradient), ‘shallow incline’ (2–8% gradient), ‘steep incline’ (>8% gradient) and ‘decline’ (<−2% gradient). These categories were adapted from those used by Parsons et al. [9]. Distance galloped from the start of each trial was recorded in metres and then divided by a factor of 100 to allow analysis of changes in stride length per 100 m galloped. Horses undertook between one and three gallops in each training session, and each different gallop was defined as a separate trial and recorded with a trial number.

### 2.4. Statistical Analysis

Stride duration was the outcome variable for the linear mixed model. Stride frequency (SF) and stride length (SL) can then be derived if required from stride duration and speed (*v*) using the following formulae:

(3) SF=1/SD

and

(4) SL= SD*v

The distributions of both stride length and stride duration were found to be normal as determined using a Shapiro-Wilk test for normality. The data were analysed using a linear mixed model for longitudinal data. Fixed effects were specified as speed, week, distance galloped per gallop, number of gallops per training session, curve, and incline. Horse ID was set as a random effect to account for multiple observations. Interactions between week and velocity, week and curve, week and incline and week and distance galloped were coded to investigate the effects of these factors over time and were included from the initial model. A quadratic factor was added for the effect of week, as this had a better fit than including week as a linear term. Following the initial model, it was possible to exclude non-significant factors from the model. The final model excluded the interactions between week and incline and week and velocity due to a lack of significance (*p* > 0.2) in the initial model. Data were stratified by age to allow comparison of all factors between groups. 

A first order auto-regressive variance-covariance structure was used to account for repeated strides from the same trial within week and horse. Residuals were found to be normally distributed and followed a non-biased homoscedastic pattern. The significance level was set at *p* = 0.05.

Statistical analysis was performed using R (R-Project for Statistical Computing, Vienna, Austria) and SAS (v9.2, SAS Institute Inc., Cary, USA).

## 3. Results

### 3.1. Two-Year-Old Horses

Data were collected over a period of 43 weeks. A total of 76,098 strides were used for data analysis. These strides were from 584 separate trials, defined as an individual gallop segment, with an average of 180 ± 92.2 strides per trial. The mean number of trials per horse per day was 1.4 ± 0.5. The mean distance galloped per trial was 925.3 m (4.6 furlongs). The mean measured speed was 11.0 ± 2.2 m s^−1^, range 7.0 m s^−1^ to 19.9 m s^−1^. The mean stride duration overall strides was 0.46 ± 0.04s and the mean stride length overall strides was 5.0 ± 0.74 m.

Horses showed a decrease in stride duration (SD) of 11.8 ms (*p* < 0.001) per 1 m s^−1^ increase in speed. There was a non-linear pattern of SD over time (*p* < 0.0001) with SD decreasing initially and then increasing quadratically over time (linear and quadratic coefficients being −0.3 ms/week and 0.006 ms/week^2^). This leads to a curvilinear relationship between the variables with a slowing rate of change over time. There was no significant interaction between week and speed. This resulted in a total decrease in SD of 12.5 ms over 43 weeks.

When speed is controlled for, galloping on a curve led to an increase in SD of 2.6 ms compared with galloping on the straight (*p* < 0.001). Over time, galloping on a curve led to a decrease in SD of 0.1 ms per week (*p* = 0.02) compared with galloping on the straight, a total decrease in SD of 3.4 ms over the 43-week trial period. Galloping on an incline or decline had a significant effect on gait. On a shallow incline (2–8%), there was a decrease in SD of 1.9 ms (*p* < 0.001). On a steep incline (>8%), there was a decrease in SD of 3.6 ms (*p* < 0.001). On a decline, there was a decrease in SD of 1.0 ms (*p* < 0.001). There were no significant interactions between week and incline (*p* = 0.09).

When controlled for speed, stride duration increased by 1.2 ms between trial 1 and trial 2 (*p* < 0.001), and by 7.0 ms between trial 1 and trial 3 (*p* < 0.001). With each 100 m galloped, horses showed an increase in SD of 2.2 ms (*p* < 0.001). Over the study period, SD decreased by 0.04 ms (*p* < 0.001) with each 100 m galloped per week. The effect of a longer gallop distance was that SD increased with distance galloped, but this effect was reduced over 43 weeks by around 1.7ms, i.e., a horse galloping a longer distance in week 43 would show a smaller increase in SD than it did in week 0.

Between-horse variation was 0.28 ms^2^. Residual variation was 0.3 ms^2^, leading to a residual interclass correlation coefficient (ρ) of 0.48. This indicates that most of the random variation is a between-horse effect, and that the random effect of horse is appropriate. A full summary of the results is shown in Table 1.

### 3.2. Three-Year-Old Horses

From the original 40 horses, twenty-four were still in training at the same yard and were available for the second year of the study. Of the horses lost to the study, one was euthanized and fifteen were sold. Six horses remained in full training over the winter period and eighteen horses had a period of light work (referred to here as ‘time off’). The data collection period for three-year-old horses spanned 34 weeks. A total of 21,072 strides were used for data analysis. These strides were from 115 separate trials, with an average of 199 ± 96.2 strides per trial. The mean number of trials was 1.3 ± 0.4. The mean distance galloped per trial was 996.8 m (5.0 furlongs). The mean speed was 11.4 ± 2.5 m s^−1^, range 7.0 ms^−1^ to 20.4 m s^−1^. The mean stride duration over all strides was 0.46 ± 0.03 ms and the mean stride length over all strides was 5.1 ± 0.9 m.

Horses showed a decrease in stride duration (SD) of 2.0 ms (*p* < 0.001) per 1 m s^−1^ increase in speed. There was no significant effect of week on SD (*p* = 0.5) or of curve on SD (*p* = 0.39). Galloping on an incline had a significant effect on gait. On a shallow incline (2–8%), there was a decrease in SD of 2.2 ms (*p* < 0.001) and on a steep incline (>8%), there was a decrease in SD of 3.9 ms (*p* < 0.001). There was no effect of decline on stride duration (*p* = 0.37). Stride duration decreased by 4.0 ms between trial 1 and trial 2 (*p* = 0.04). With each 100 m galloped, horses showed an increase in SD of 2.0 ms (*p* < 0.001).

Overall, there was no difference in SD between horses that had a period of ‘time off’ over the winter compared to those that did not have any time off (*p* = 0.90). However, horses that had a period of time off showed a decrease in SD of −0.6 ms per week (*p* = 0.02) over time compared with those that did not have time off. This indicates a more rapid rate of change in comparison to the two-year-old horses. Horses that had a period of time off also showed a significant interaction between time off and distance galloped, and showed an increase in stride duration of 1.1 ms per 100 m galloped (*p* = 0.01) compared to horses that did not have time off.

Between-horse variation was 0.2 ms^2^ and residual variation was 0.7 ms^2^, ρ = 0.2. A full summary of the results is shown in Table 2.

## 4. Discussion

### 4.1. Gait Changes in Response to Training

In this cohort of horses, we found a total decrease in stride duration of 12.5 ms over a 43-week period during their two-year-old training season. For a horse galloping on the flat, in a straight line, at the start of a trial, at 11 m s^−1^ with a stride duration of 541.2 ms (based on measurements from week 0 of data collection), this represents a 2.3% change in stride duration. Overall, no significant change to stride duration was seen in three-year-old horses. However, those horses that had a period of rest or low-intensity training over the winter months demonstrated a similar change in stride duration to untrained horses compared to horses that had no time off. For a horse that had time off and then returned to full training, galloping on the flat in a straight line at 11 m s^−1^, a 4.2% change in stride duration of would occur over the 34-week data collection period. This represents a smaller change, which occurred at a faster rate, suggesting that the effect of training or residual fitness gained in the two-year-old season was retained. 

The effects of maturation in addition to training on stride parameters in the two-year-old group of horses cannot be ruled out. Minor conformational changes take place between the ages of two and three, with an increase in the angle of the scapular spine and a decrease in hoof length [17]. Due to the nature of the study using Thoroughbreds in real-world conditions, the training effect cannot be fully separated from other factors such as growth or fitness.

Previous studies of galloping horses have described conflicting results. On a treadmill at the canter Thoroughbred fillies demonstrate a decrease in stance duration with no change in stride length or frequency [14], and polo ponies demonstrate an increase in stride duration [18]. Mature Thoroughbreds training overground show an increase in stride frequency with no change in stance duration but a decrease in protraction time with training [6]. The study techniques are likely to explain these differences, as during treadmill locomotion horses tend to increase stride duration and stance duration in relation to locomotion over ground [19].

In humans, elite runners display a higher stride frequency and a shorter stride length compared to less trained runners [20] and an increased stride length and decreased stride frequency is associated with an increase in impact force [21,22]. Given the association between impact forces and injury [23,24], the reason for this adaptation is unclear.

### 4.2. Changes in Gait in Response to Environmental Factors and Training

When galloping on a curve, the horses showed an increase in SD of 2.61 ms compared with running on the straight. This is consistent with the theory that in horses force limits speed when running on a bend [13], assuming that the longer SD is caused by an increase in stance duration rather than an increase in protraction duration as protraction duration is typically consistent at different speeds [11,12], and when turning at the walk horses increase stance duration [25]. The overall decrease in SD with time suggests that with training, gait change is seen that results in the horse running on a curve in a more similar fashion to running on the straight. This may represent a ‘learning response’, or perhaps neuromuscular adaptation to training [6,7].

In the three-year-old horses, an increase in SD of 0.73 ms was seen, but this was not significant. Given that there appears to be no significant effect of curve running on SD in the three-year-old cohort of horses, this suggests that over time horses may be able to adapt to curve running. This is contrary to what might be expected, as it is likely that force limits curve running [13], and therefore at a given speed, it would be expected for a horse to demonstrate a longer stride duration in order to reduce maximal loading. However, as horses training are typically performing at submaximal speeds, it is possible that this force limitation is not a factor in determining gait adaptations, and that learning effects are more relevant here.

Horses decrease SD when galloping on an incline, which is likely due to a concurrent decrease in protraction duration during incline running [9]. This may be attributed to either the interruption of the protraction arc of the limb by the surface, or a higher level of energy storage in the limb’s ‘catapult’ protraction mechanism [9]. On a decline, the horses in this study again showed a decrease in SD. The reasons for this are less clear, as previous work in the walk and trot has shown an increase in SD and SL when moving downhill [26] and that horses slow down when racing downhill [8]. Further work to establish specifics of gait change on a decline is required. There was no significant effect of week of training on incline running. This lack of change suggests that adaptations to incline/decline galloping may be more constrained than galloping on a curve.

With each extra 100 m galloped, horses displayed an increase in SD in both age groups which is likely to be a response to fatigue, as an increase in stride duration due to fatigue occurs in horses [27,28,29,30], humans [31] and guinea fowl [32]. Over time, gait change in response to distance galloped decreases, suggesting that the horses are getting fitter. With increased fitness, aerobic capacity is increased and muscular adenosine diphosphate (ADP) increases, leading to a decrease in SF [33] and consequently a decrease in SD.

### 4.3. Biomechanical and Musculoskeletal Factors

From a biomechanical perspective, we often expect animals to move in the most efficient way, which may explain in part the adaptations seen here. For example, when allowed to select their own speed within a gait, horses will automatically choose the most energetically efficient speed [34]. As the cost of locomotion is associated with the cost of activating muscle and generating force to move the limbs, stride frequency is directly related to the cost of locomotion. When comparing between quadrupedal mammals, the cost of locomotion per kilo per stride at an equivalent speed is the same [35], so it might be expected that longer strides are more energetically efficient. However, considering the collisional theory of running, it is more energetically efficient to take shorter steps and minimise the distance that the centre of mass (COM) falls during each step, as re-directing the COM back into an upward trajectory is energetically costly [36]. It has been suggested that collisional losses could be vastly reduced by taking very small steps, although additional work would then have to be done to swing the leg [37]. Bertram [37] proposes that the minimum cost of gait falls in between long steps with a loss of energy from collisions and short steps with a loss of energy from swing.

Due to the large cohort of horses in a commercial setting and concurrent necessary simplification of data collection, this study is limited by the inability to conclusively differentiate between changes in stance or swing time to explain further the gait changes seen in this study. Based on previous work [7,14,38,39], we postulate that the main changes are likely to be due to changes in the stance phase of the gait, although based on studies of older horses [6] it is impossible to rule out a component of swing phase change. If we assume the gait changes seen here are due to a change in stance duration, for a typical stance of 110ms at 11 m s^−1^ [12], there is a change of 11.3% over the 43-week period. This would lead to an increase in peak vertical GRF of approximately 5.2 N kg^−1^ for a typical Thoroughbred galloping at 11 m s^−1^ following training.

If any of the stride duration change is due to alterations in the swing phase of the stride, this is likely due to changes in the mechanics of the limb over time. The energy-storing superficial digital flexor tendon (SDFT) increases its cross-sectional area (CSA) with training in juvenile animals [40,41] but it is generally considered that energy-storing tendons do not adapt after around two years of age [42]. An increase in tendon CSA will lead to increased stiffness [43,44], which will alter the frequency of the spring-mass system of the limb and therefore an increase in energy-storing capacity, assuming that an increased force is applied. The *Biceps brachii* muscle also appears to have an energy-storing role [11], so the same effect is likely seen in the swing phase of the stride.

Increased fitness is likely to play a role in the gait changes observed. During a training program, muscle mass increases and muscle composition changes, with a decrease in type IIb muscle fibres [45] and an increase in type IIa and type IIbx muscle fibres [46]. In addition, biochemical changes take place, indicating an increase in oxidative capacity and an increase in strength of the muscles, which may play a role in limb protraction [45,46]. An increase in metabolic capacity of the muscles would delay fatigue and would help explain the decrease in stride duration with distance galloped seen over time, in addition to the overall decrease in stride duration seen over time. Given that horses have a largely passive mechanism for protracting the forelimb [11], we also suggest that in the Thoroughbred, an increase in stride frequency at the expense of an overall shortened stride may maximise the energetic return from the passive limb protraction mechanism. Fatigue increases injury risk [28,47] via increases in bone strain [48], muscle force variability [32] or an increase in impact at foot on and increased likelihood of slipping [49]. Therefore, adaptations to minimise fatigue responses are likely beneficial to the individual, and an understanding of normal responses during training is therefore important.

Regardless of the mechanism causing a decreased stride duration in Thoroughbred horses, if this is due to any decrease in stance duration there is likely to be a trade-off between increases in energetic efficiency and the increased forces experienced by the distal limb. Limb force is likely to be a limiting factor in maximal equine performance, [8,12,13] in addition to changes in force altering injury risk. 

This study provides an insight into changes to stride parameters with training. The relatively small cohort and use of convenience sampling at one stable represents a significant limitation as inter-trainer effects cannot be evaluated. The clinical significance of the gait changes observed here is unknown, and the study provides baseline data for future comparisons.

Increasingly simple and accessible measures of gait and performance are becoming available for trainers, and a knowledge of normal adaptations to training is crucial to allow these technologies to be exploited to their maximum. This study supports the hypothesis that Thoroughbred horses undergoing race training show a decrease in stride duration over time and demonstrates normal changes that can be monitored using a single sensor. Horses demonstrate reduced gait alteration in response to galloping over longer distances over time, which is likely fitness-related. Horses demonstrate some adaptation to curve running over time, but there is no alteration in SD over time for incline running, suggesting that adaptations to running uphill are more constrained. 

## Figures and Tables

**Table 1:** Final multivariable model for fixed effects on stride duration (ms) for two-year-old horses. Data were 76,098 strides collected over 584 separate gallop trials from 40 horses over 43 weeks. Reference categories for incline and curve are flat and straight respectively. Week * week indicates the quadratic factor.

Effect	Category	Stride Duration (ms)		
		Estimate	Standard Error	*p*-Value
Speed		−11.78	0.06	<0.001
Week		−0.29	0.05	<0.001
Week * week		0.006	0.001	<0.001
Incline Category	Flat	0		
	Shallow incline	1.89	0.24	<0.001
	Steep incline	−3.62	0.44	<0.001
	Decline	−1.10	0.29	<0.001
Curve Category	Straight	0		
	Curve	2.61	0.69	<0.001
Trial number	1	0		
	2	1.21	0.31	<0.001
	3	7.00	0.96	<0.001
Distance galloped (per 100 m)		2.21	0.007	<0.001
Interaction between week & distance galloped		−0.04	3.3 × 10^−6^	<0.001
Interaction between week & curve category	Curve	−0.08	0.03	0.020

**Table 2:** Final multivariable model for fixed effects on stride duration (ms) for three-year-old horses. Data were 21,072 strides from 115 separate gallop trials from 24 horses over 34 weeks. Reference categories for incline and curve are flat and straight respectively, and the reference category for time off is no time off. Week * week indicates the quadratic factor.

Effect	Category	Stride Duration (ms)		
		Estimate	Standard Error	*p*-Value
Velocity		−1.95	0.20	<0.001
Week		−0.05	0.08	0.52
Incline Category	Flat	0		
	Shallow incline	−2.17 *	0.49	<0.001
	Steep incline	−3.92 *	0.85	<0.001
	Decline	−0.55	0.61	0.37
Curve Category	Straight	0		
	Curve	0.73	0.86	0.39
Trial number	1	0		
	2	−4.00 *	1.92	0.04
Distance galloped (per 100 m)		1.99 *	0.19	<0.001
Time Off	No	0		
	Yes	1.03	7.87	0.90
Interaction between time off & week	No time off	0		
	Time off	−0.59 *	0.24	0.02
Interaction between time off & distance galloped	No time off	0		
	Time off	1.05 *	0.41	0.01
